# Rapid Detection of Virus Nucleic Acid via Isothermal Amplification on Plasmonic Enhanced Digitizing Biosensor

**DOI:** 10.3390/bios12020075

**Published:** 2022-01-28

**Authors:** Shih-Chung Wei, Chia-Chen Chang, Tsung-Liang Chuang, Kung-Bin Sung, Chii-Wann Lin

**Affiliations:** 1 Institute of Biomedical Electronics and Bioinformatics, National Taiwan University, Taipei 10617, Taiwan; wshihchung@gmail.com (S.-C.W.); kbsung@ntu.edu.tw (K.-B.S.); 2 Institute of Biomedical Engineering, National Taiwan University, Taipei 10617, Taiwan; light02062005@gmail.com; 3 Department of Medical Biotechnology and Laboratory Science, Chang Gung University, Taoyuan 33302, Taiwan; chang@mail.cgu.edu.tw; 4 Kidney Research Center, Department of Nephrology, Chang Gung Memorial Hospital, Taoyuan 33302, Taiwan; 5 Biomedical Technology and Device Research Laboratories, Industrial Technology Research Institute, Hsinchu 31057, Taiwan

**Keywords:** LAMP, plasmonic, nanoarray

## Abstract

Rapid detection for infectious diseases is highly demanded in diagnosis and infection prevention. In this work, we introduced a plasmonic enhanced digitizing biosensor for the rapid detection of nucleic acids. The sensor successfully achieved the detection of loop-mediated isothermal amplification for the hepatitis virus in this work. The sensor comprised a nanodisc array and Bst polymerases conjugated on the rough surface of a nanodisc. The rough surface of the nanodisc provided plasmonic hot spots to enhance the fluorescence signal. The virus DNA was detected by conducting a modified loop-mediated isothermal amplification with fluorescence resonance energy transfer reporter conjugated primers on the sensor. The modified isothermal amplification improved the signal contrast and detection time compared to the original assay. By integrating the modified amplification assay and plasmonic enhancement sensor, we achieved rapid detection of the hepatitis virus. Nucleic acid with a concentration of 10^−3^ to 10^−4^ mg/mL was detected within a few minutes by our design. Our digitizing plasmonic nanoarray biosensor also showed 20–30 min earlier detection compared to conventional loop-mediated isothermal amplification sensors.

## 1. Introduction

Rapid and accurate pathogen screening technology is important for the early diagnosis and prevention of infectious diseases. Plasmonic resonance is one of the most important technologies in biosensor development [1], especially in plasmonic-enhanced biosensors, for example, fiber optics surface plasmon resonance (FO-SPR) [2,3], plasmonic enhanced fluorescence [4] and surface-enhanced Raman spectroscopy [5,6]. The enhancement of fluorescence and Raman is based on the increase of the local electric field on the surface of the nanostructure. The increase in the local electric field is caused by a localized surface plasmon resonance and lightning rod effect. When the excitation light is close to the resonance frequency of the nanostructures, the localized surface plasmon and lightning rod effect will generate a strong local electric field on the structure surface and the edge of the structure [7]. This plasmonic enhancement technology has been used in DNA hybridization detection [8], end point DNA amplification detection [6] and time series DNA amplification monitoring [9].

Loop-mediated isothermal amplification (LAMP) is one of the widely used isothermal technologies for rapid pathogen DNA detection [10]. In the first step of the reaction, with a special primer design, the amplicons have two self-annealing ends to form dumbbell-like new templates. In the second step, this self-annealing dumbbell-like template proceeds the elongation step with the help of additional primers to form flower-like products. With the DNA unwinding function of Bst polymerase (*Bacillus stearothermophilus*), rapid detection was achieved by skipping the precise thermal cycle for unwinding double-stranded DNA in conventional PCR [11]. Due to the advantages of rapidity, sensitivity and specificity, LAMP has been used in human pathogen detection [12]. Different designs and devices have been developed to further extend the application and simplify the experimental procedures [13], for example, multiplex LAMP [14], droplet microfluidics [15,16], microfluidic chambers [17,18], optical fiber integrated microfluidics [19], surface plasmon resonance [20] and integrated microfluidics [21].

Real-time LAMP monitoring is usually based on fluorescent intercalation dye [22], solution turbidity [23,24] or refractive index change [25]. To improve the sensing specificity, Tani et al. introduced a reporter-conjugated primer design and alternatively binding quenching probe competition assay to LAMP [26]. In 2011, Kubota et al. described an assimilating probe design for LAMP based on fluorescence resonance energy transfer (FRET-LAMP) and demonstrated the real-time detection with sequence specificity [27]. This assay has also been used in pathogen detection; for example, Chou et al. demonstrated the detection of white spot syndrome virus of penaeid shrimp [28]. Generally, FRET-LAMP is a specific and sensitive nucleic acid assay without a post-amplification process and cross-contamination [29]. Moreover, FRET-LAMP has also shown its capability in multiplexing [30]. However, all these LAMP technologies still require 20–40 min for nucleic acid detection.

In this article, we combined the FRET-LAMP with a nanoarray and digitizing method to achieve rapid isothermal nucleic acid detection. In our previous study, single-spot LAMP with signal enhancement achieved the rapid detection of virus DNA [9]. However, fluctuations of the reaction are a serious concern in single-spot reactions, which might come from the molecular transportation and collision frequency problem [31]. Hence, a new strategy of using a nanoarray for parallel FRET-LAMP was conducted to overcome this problem. Counting the number of reaction spots in micro- [32] or nanoarray biosensors [33] has been reported to increase the sensitivity and improve the detection limit of immunoassays [34] and LAMP [35] compared to acquiring the fluorescence intensity. In this work, we successfully demonstrated an improvement in the signal contrast and detection time of LAMP with this novel detection strategy. Compared to other plasmonic enhanced sensors, the FRET array-LAMP provided a time series monitoring platform and digitizing analysis to achieve rapid pathogen DNA detection.

## 2. Materials and Methods

### 2.1. Materials 

The etching solution for nanoarray manufacturing was composed of 0.076-g thiourea (≥99%, Sigma-Aldrich Co., St. Louis, MO, USA), 0.2686-g ferric nitrate (≥98%, Sigma-Aldrich Co., St. Louis, MO, USA) and 18-μL 1-octanol (≥99%, Sigma-Aldrich Co., St. Louis, MO, USA) in 50-mL water. The linker used for nanostructure surface functionalization was 16-mercaptohexadecanoic acid (MHA, 9%, Sigma-Aldrich Co., St. Louis, MO, USA), which was activated by 1-ethyl-3-(3-dimethylaminopropyl) carbodiimide (EDC, >98%, Sigma-Aldrich Co., St. Louis, MO, USA) and N-hydroxysuccinimide (NHS, 98%, Sigma-Aldrich Co., St. Louis, MO, USA). The LAMP reagent mixture included 0.4-mM dNTPs, 0.4-M Mg2SO4, 1-M betaine (≥99%, Sigma-Aldrich Co., St. Louis, MO, USA), 1×Bst buffer (New England Biolabs Inc., Ipswich, MA, USA) and the primer mixture (synthesized by PURIGO Biotechnology Co., Taipei, Taiwan) in ultra-pure water. The primer mixture contained 10 pmol of the forward/backward primers (FP/BP), 5 pmol of the forward/backward loop primers (LF/LB), 20 pmol of the backward inner primer (BIP) and 20 pmol of the forward inner primer (FIP). All primer designs were based on our previous publications [9,36]. For FRET-LAMP, the FRET primer pair was used instead of FIP. The FRET primer pair was composed of a fluorophore reporter conjugated FIP (F-FIP) and a quencher conjugated complementary oligonucleotide probe (Q-FIPc) which are listed in Table 1. For multiplex detection, each FRET primer pair of two different targets was a half-concentration compared to the conventional LAMP to make the total primer concentration consistent between all the experiments in this article. The Bst polymerase (76 kDa) used was from New England Biolabs Inc. All the hepatitis DNA/cDNA templates were from the stock plasmid prepared by Dr. Lee in his previous work. Gene fragments were amplified and cloned into the pGEM-T Easy Vector system (Promega, Madison, WI, USA) for long-term storage. All the templates used in this research were from the storage plasmid pool without any pathogenesis. All the solid and liquid wastes were disposed as biohazard wastes.

### 2.2. Preparation of Tip and Nanoarray for LAMP

A metallic tip and nanodisc array were prepared for single-spot and array LAMP, respectively. For-single spot LAMP, a gold film of seven nm thick was deposited onto the Pt/Ir-coated tips (NanoInk Inc., Skokie, IL, USA) by sputtering (Figure S1). The diameter of the tip apex increased from 30 nm to 55 nm after sputtering. Biomolecule modification followed the methods mentioned in our previous work [9]. Briefly, a self-assembled monolayer of MHA was formed at the gold film surface by a thiol-metal bond (Au-S bond). The MHA was then activated with freshly prepared 400-mM EDC and 100-mM NHS by soaking into EDC/NHS solution for 5 min. Bst polymerase was then functionalized on the tip apex by approaching the tip of the polymerase-contained cellulose membrane. The amine group on the polymerase was covalently bound to the carboxyl group of the MHA. The functionalized tip was then used for LAMP immediately.

For array-LAMP, a gold nanodisc array was produced by the dip-pen nanolithography platform (DPN, NanoInk Inc., Skokie, IL, USA). Gold film was deposit on clean glass slides by sputtering as a substrate. The metallic tips were dipped into MHA solution and used to draw patterns on the gold film [37]. The MHA molecules spontaneously assembled on the gold film surface and formed a self-assembled monolayer (SAM). The thiol end of MHA was linked to the gold film surface via thiol-metal bonding (Au-S bond) and worked as a resistant mask and linker to the biomolecules [38]. The whole chip with a resistant mask pattern was then immersed in etching solution (0.076-g thiourea, 0.2686-g ferric nitrite and 18-μL 1-octanol in 50-mL water). The pH value of the etching solution was preadjusted to 1.85 by adding HCl. After 15 min of etching, the chip was washed in an ultrasonic water bath. After washing, the array was activated with EDC/NHS solution following the same protocol as single-spot LAMP. A drop of Bst polymerase was dropped onto the array and stayed for five min for protein functionalization.

The distance between the metal structure and the fluorophore was estimated to assess the possible quenching caused by the metal surface (Figure S2). The length of the MHA linker was around 1–1.5 nm, and the diameter of Bst polymerase was 5.6 nm, while the shape of the polymerase was assumed to be spherical [39]. Thus, the distance between the gold and fluorophore was estimated to be 6.6–7.1 nm. The distance was thought to have fluorescence enhancement [40,41].

### 2.3. FRET-LAMP of Hepatitis C Virus and Hepatitis B Virus

In the first stage of LAMP, the amplicon initiated with FIP and BIP formed dumbbell-like DNA loops, which was the new template for further amplification. The new template then replicated into a flower-like long-chain DNA strand by self-annealing [42]. In addition, loop primers were used to accelerate the amplification process. The primer sequences for HCV and HBV were the same as those reported previously [9,36]. 6-carboxyfluorescein (6-FAM) with the maximum emission in 520 nm was conjugated to the 5′ end of HCV FIP, and carboxytetramethylrhodamine (TAMRA) with the maximum emission at 576 nm was used for HBV FIP instead. A nonfluorescent quencher (NFQ) with an effective absorbance ranging from 390 to 625 nm was linked to the 3′ end of both the Q-FIPc for HCV and HBV. The Q-FIPc was prehybridized to F-FIP overnight before using. For performing the LAMP reaction, the LAMP reagent mixture in Section 2.1 was dropped into reaction chambers with tips or the nanoarray inside. The chambers were then sealed and heated to 65 °C. The fluorescence signal was acquired by a homebuilt two-photon microscope [9], and the illumination power was measured after the laser intensity attenuator. According to our design, each newly formed double-stranded DNA initiated with F-FIP has one fluorophore at one of the two ends.

## 3. Results

A nanoarray functionalized with Bst polymerase was designed for fast detection LAMP with a fluorescence resonance energy transfer primer. By digitizing the reaction spot, we could monitor the process of DNA amplification and observe the early increase of the amplification signal compared to the conventional method. A gold disc nanoarray was fabricated by DPN and functionalized the array with Bst polymerases, as shown in Figure 1①. We used MHA as the ink, which is a long-chain molecule with thiol and carboxylic acid in two ends, respectively, for the DPN fabrication. We then drew the designed nanoarray pattern with the ink on a gold film. To prevent the gold film from peeling during the whole process, we deposited 5-nm titanium as an adhesion layer on the glass slide by E-gun before sputtering 40-nm gold film. The thiol end of MHA formed an Au-S bond with the gold film. This MHA layer could be a resistant layer and protect the gold under MHA from etching. After the etching step, the gold protected by MHA remained on the glass slide and became a nanodisc array. The other functional group end of MHA on the gold nanodisc, which is carboxylic acid, could be a linker connected to the target molecules. By activating with EDC/NHS, the MHA molecule formed an amide bond with an amine group on Bst polymerase. This functional nanoarray would be used immediately for the LAMP reaction after fabrication. For following the LAMP reaction, the nanoarray was surrounded with a hollow-squared double-sided tap to form a chamber. The chamber was sealed with a coverslip after adding the FRET-LAMP reagent and target DNA template. The reaction was then carried out at the heating stage of a two-photon microscope for time lapse observation. The templates would be amplified by Bst polymerase on the nanodisc once the chip was heated to 65 °C.

Any replicon starting with a FIP primer always has one fluorescence reporter at the 5′ end, as shown in Figure 1②. During the FRET-LAMP reaction, digitized counting of those bright spots on the nanoarray was performed, as shown in Figure 1③, and then, the number of bright spots was recorded to illustrate the reaction trend with the time (Figure 1④).

### 3.1. Au Nanostructure for Electromagnetic Filed Enhancement

**Figure 2:**
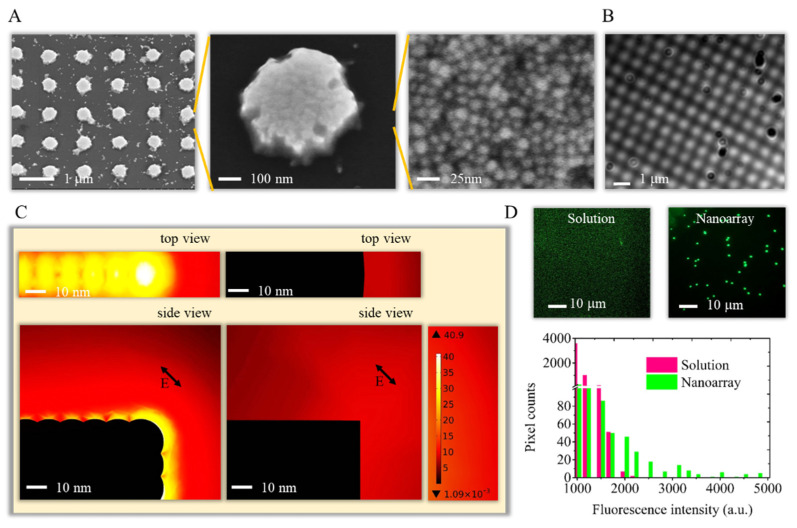
(**A**) The nanodisc was investigated with a scanning electron microscope. (**B**) The scattering image of the nanodisc array was recorded by a dark-filed microscope. (**C**) The near-field electrical field of the nanodisc was simulated with COMSOL Multiphysics. The rough surface provides a higher electrical field enhancement than the smooth surface. (**D**) The image and histogram of the fluorescence signal from LAMP in the solution and nanodisc. The LAMP reaction on the nanodisc showed brighter signal than the reaction in the solution.

A nanobiosensor with plasmonic enhanced fluorescence was designed for digitizing sensing. To achieve signal enhancement, nanodiscs with rough surfaces were fabricated with dip-pen nanolithography and chemical etching. The tips for dip-pen nanolithography were soaked in MHA dissolved in pure acetonitrile for carrying writing inks. The ink was then transferred to the surface of a pre-sputtered Au film on a glass substrate by the water meniscus between the tip and surface during contacting. After etching, a nanodisc array with a 400-nm diameter and 40-nm height was then generated with the MHA ink on the gold surface. The center-to-center distance of the dots was one micrometer to prevent electrical field interference between structures, as shown in Figure 2A. The etching process generated a rough surface of the nanodisc because of the detachment of gold grains that accumulated on the glass during sputtering. The size of these grains was around 5–20 nm in diameter under a scanning electron microscope. The incident light could interact with this nanodisc structure to generate a concentrated electrical field on the near surface on the nanodisc and then scattering out. The scattering of the nanodisc array was observed with a dark-field microscope (Figure 2B).

The concentration or enhancement of the electrical field on the metal surface is related to the material, shape and roughness of the metal structure. We simulated the intensity of the electrical field on the nanodisc with COMSOL Multiphysics to demonstrate the contribution of this roughness to the enhancement of the optical intensity (Figure 2C). An incident electromagnetic plane wave at 800 nm shone at 45 degrees, representing one of the focusing rays of the laser on the structure. The initial electrical field intensity (E0) was set at 1 V/m. The structure material setting was gold, and the surrounding environment setting was air. The intensity of the electromagnetic wave was set as one. According to the simulated electrical field intensity map, a gold nanodisc with a smooth surface could enhance the intensity of the electrical field only around 10–15 times at the edge of the disc. Impressively, with the roughness caused by gold grains, the enhancement of the electrical field elevated to 30–45 times compared to the incident light.

To demonstrate the enhancement of the fluorescence signal for the LAMP reaction, we compared the fluorescence intensity of the reactions in the solution and on the nanodisc structure (Figure 2D). The fluorescence images showed a brighter reaction on the nanoarray than the reaction in the solution. The histogram of the images clearly indicated a higher pixel count in the nanoarray for pixel intensities higher than 2000. The reasons for the signal intensity increasing could be the enhancement of the optical intensity by the gold nanostructure and condensed reactions at the surfaces of the nanodiscs.

### 3.2. FRET-LAMP for Amplifying Hepatitis Virus Nucleic Acid

**Figure 3:**
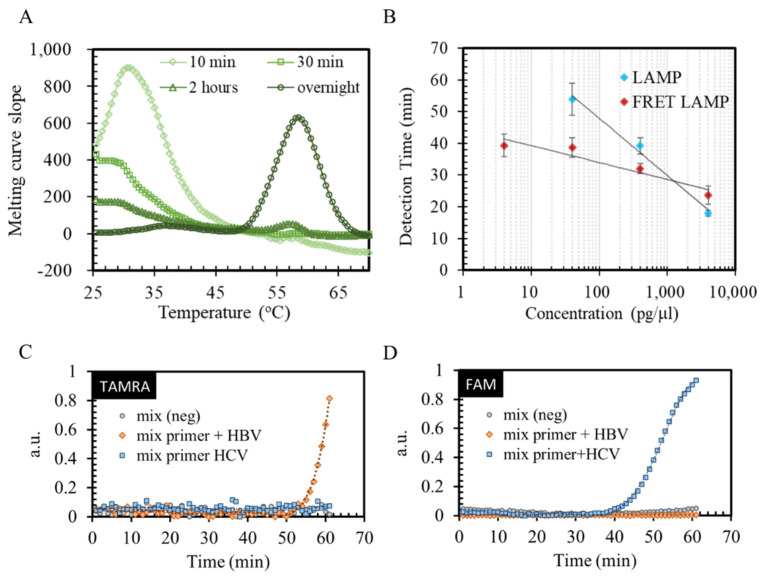
(**A**) Melting curve was used to investigate the hybridization of the fluorophore primer and quencher probe. The results indicated that the FRET primer pair required overnight incubation under 4 °C to achieve the designed melting temperature. Hence, all the FRET-LAMP in our experiment used the FRET primer pair after overnight incubation. (**B**) The detection time calibration curve of conventional LAMP and FRET-LAMP. FRET-LAMP provided a better detection limitation and detection time of real-time LAMP for template concentrations lower than 400 pg/μL. (**C**,**D**) The real-time fluorescence curve of FRET-LAMP revealed the capability for multiplex detection. The specificity of the amplification was verified with two different emission channels (mix primer: mixture of the HCV and HBV primer sets).

Conventional LAMP for HCV and HBV were performed by the real-time PCR detection system BIO-RAD CFX (BIO-RAD, Hercules, CA, USA) for primer design verification. Before applying to the reaction, a synthesized FRET primer pair was investigated by the melting curve to ensure that the primer pair hybridized properly. The primer pair solutions were heated from 25 °C to 90 °C gradually, and the change of the fluorescence signal was recorded. During the heating process, the increase of the fluorescence intensity reflected the unwinding status of the primer pair. The peak of the fluorescence intensity slope in Figure 3A indicates the temperature point with a dramatic increasing of the fluorescence. By comparing the peak of the slope, the results indicated that overnight incubation was necessary for the primer pair to hybridize properly and reach the designed melting point at 57 °C. Well-hybridized primer pairs were then used in the real-time LAMP (Figure 3B). To investigate the amplification response to the target number, different concentrations of HCV cDNA were spiked into the reaction buffer. The time points when the reaction signal exceeded a threshold intensity are plotted in Figure 3B. The threshold was set around the beginning of the log-linear phase of the intensity change of the amplification process. The results suggested that the FRET-LAMP for HCV could shorten the detection time and provide better detection of the limit. The signal was observed after 40 min in FRET-LAMP for a template concentration at 40 pg/μL, which was 10 min earlier than the conventional LAMP. In addition, under the same reaction conditions, the lowest detection concentration of FRET-LAMP was 4 pg/μL, while conventional LAMP showed no signal within 60 min. The detection limit of FRET-LMAP was 10 times better than conventional LAMP for this reaction condition. The detection time here was defined as the time when the reaction curve exceeded the threshold and entered a log-linear phase.

A multiplex detection for HCV and HBV was also demonstrated in Figure 3C,D. Either HCV, HBV or pure water reagent was added into the reagent–primer mixture for real-time FRET-LAMP. Different colors of fluorophore were used to identify two different virus nucleic acids. For HBV, the FRET primer was conjugated with TAMRA fluorophore; for HCV, the FRET primer was conjugated with FAM. The signal of the HBV DNA-spiked sample only increased in the TAMRA channel, while the signal of the sample spiked with HCV cDNA only increased in the FAM channel. The results revealed the capability of FRET-LAMP for multiplex detection and showed the specificity in multiplex detection. As the results showed, the signals in each channel were increased only when the correct templates were used. There was no crosstalk between these two FRET-LAMP reactions. The results of FRET-LAMP were verified with gel electrophoresis (Figure S3).

### 3.3. Signal Contrast Investigation with Single-Spot LAMP

**Figure 4:**
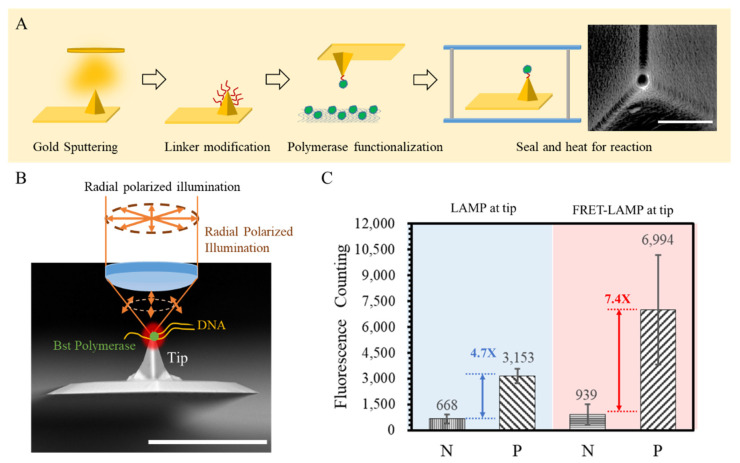
(**A**) An illustration of the single-spot LAMP preparations. A thin film of gold was deposited on the tip. The gold surface was then modified with thio-linkers for protein conjugation. Bst polymerases were functionalized on the tip through a “fishing” procedure by touching the tip to a cellulose membrane pre-immersed with Bst polymerases. The functionalized tip was then sealed in a heating chamber under microscope for reaction observations. (**B**) The illustration of the excitation illumination of single-spot LAMP (scale bar is 30 μm). Radial polarized illumination was used to generate better plasmonic enhanced fluorescence. (**C**) The signal contrast for single-point LAMP could be increased by using a FRET primer design instead of conventional SYBR dye under 30-mW illumination (N of conventional LAMP is 5; N of FRET-LAMP is 2).

Single-spot LAMP was performed to investigate the contrast improvement of FRET-LAMP compared to conventional LAMP. Figure 4A illustrates the process of performing a single-spot LAMP reaction. Bst polymerases for the LAMP reaction were coated on the apex of the tip by a MHA linker between the enzyme and a gold film deposited tip. To confine the enzyme in the apex of the tip, the approaching function of the AFM instrument was used to automatically contact the tip with a cellulose membrane containing Bst polymerase. The SEM image of the functionalized tip is shown in Figure 4A. The fluorophore and gold tip were separated by the distance of the linker and polymerase size, which could maintain enough distance for an enhancing effect. The functionalized tip would be sealed in a glass chamber prefilled with reagent for the LAMP reaction. The chamber was then heated up to 65 °C by a stage heater. To monitor the reaction, a radial polarized laser beam was used to scan the fluorescence image of the apex for fluorescence excitation, as shown in Figure 4B.

Based on this single-spot LAMP technology, we performed endpoint detection for FRET single-spot LAMP and original single-spot LAMP with SYBR^®^ Green (Figure 4C) and compared the endpoint signals of the positive and negative results under 30-mW illumination. Fluorescence photon counting was acquired after 80-min reacting. Since different fluorophores were used for FRET single-spot LAMP and original single-spot LAMP, the comparison focused on the signal contrast between the positive and negative samples instead of the absolute intensity. According to the results, FRET single-spot LAMP provided a higher contrast between the negative and positive reactions. The intensity ratio between the positive and negative reactions in FRET single-spot LAMP was 7.4-fold under 30-mW illumination, while single-spot LAMP with SYBR^®^ Green was only 4.7-fold. The results suggested that the complement probe with a quencher effectively inhibited the background signal from those fluorophore primers that were not involved in the amplification process. Compared to SYBR^®^ Green, the FRET primer pair could provide a better contrast to the background.

### 3.4. Digitizing Counting for FRET Array-LAMP

**Figure 5:**
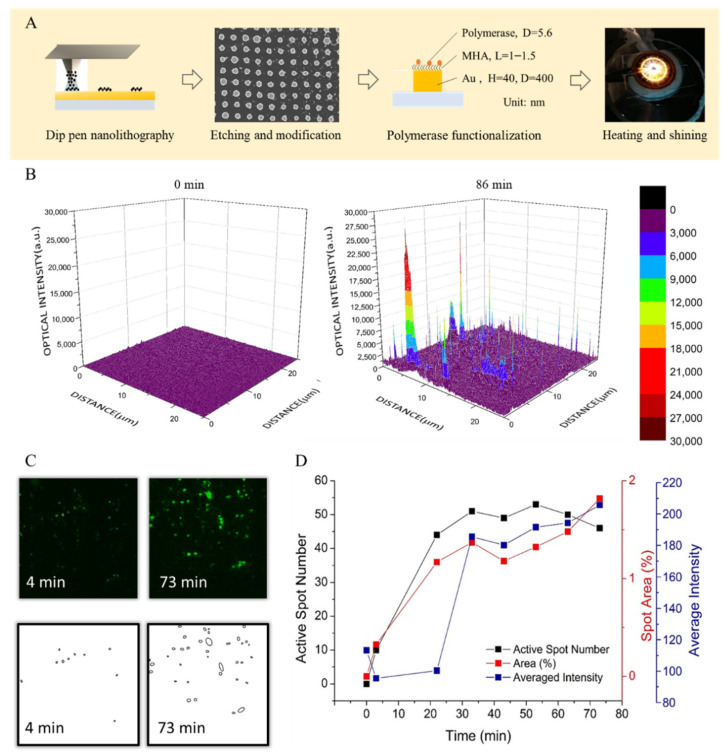
(**A**) The illustration of the array-LAMP procedure. The array was fabricated with dip-pen nanolithography with an MHA ink. After pattern etching, Bst polymerases for the LAMP reaction were conjugated on the nanodiscs. The array and reaction reagent were then sealed in a heating chamber above the microscope for observation. The amplicons with fluorophore were expected to be only a few nanometers away from the nanodisc, which was still within the plasmonic electrical field to enhance the fluorescence signal. (**B**) The fluorescence intensity map of FRET array-LAMP for the HCV gene at different time points. The fluorescence signal on the array was acquired with two photon microscopies in a scanning manner at time zero and the end of amplification. The heat maps were generated by Origin. (**C**) To identify nanodiscs with amplicon, the fluorescence images were transformed into binary images based on a preset threshold. The number of positive nanodiscs were then counted with the build-in function in ImageJ. (**D**) Counting number, bright areas and photon counting values of the same array-LAMP were compared in a time series to indicate the differences in detection times by using different analysis methods. (The template concentration was 4 ng/μL.).

A 20 by 20 nanodisc array was used for the FRET array-LAMP. Figure 5A illustrates the process of array-LAMP, from array manufacturing to LAMP reaction. The array used in this research was made by dip-pen nanolithography. The writing pen was dipped into MHA ink solution for writing an array mask on a gold film. The gold without a MHA mask was dissolved. The SEM image of the array is shown in Figure 5A. The radius of each nanodisc of the array was around 200 nm, and the center-to-center distance between discs was 1 μm. The distance between discs was slightly larger than the optical limitation, which was suitable for fluorescence imaging. The MHA mask was further activated by EDC/NHS for functionalizing Bst polymerase. For array-LAMP, the reagent was dropped on the array. The chip was sealed with cover glasses. The reaction was carried out on a microscope with a stage heater for monitoring.

To monitor the LAMP reaction, we scanned the array with a two-photon microscope and acquired the fluorescence image. Figure 5B is the fluorescence intensity mapping of the FRET array-LAMP for HCV. The map on the left is the fluorescence intensity at the start of the heating. The map on the right shows the increase of the intensity after an 86-min reaction. The peaks in the plot indicate those spots with DNA amplification. To further investigate the process of amplification, we analyzed the fluorescence intensity map at different time points based on the total fluorescence intensity, lit-up area and the number of bright spots. For determining the fluorescence intensity, we added up all the pixel values in one map and averaged the total pixel number. For calculating the lit-up area and counting the bright spots number, we transformed the intensity map into a binary image after subtracting to get a fixed threshold value. The threshold was set as one thousand photon counts based on three standard deviations above the mean mapping value of a blank sample. This threshold was applied to all the array mapping images for subtracting the background. The new images were then transformed into binary for counting the spot numbers and calculating the spot areas by ImageJ (Figure 5C). The spot area was defined as the proportion of (non-zero) pixel numbers in the binary image. The final results were plotted in Figure 5D. The detection time by the fluorescence intensity was similar to the consequence of FRET-LAMP in Figure 3. The detection time was defined as the time before the signal reached a plateau, which was estimated to be 5–20 min for the active spot number or spot area and 25–30 min for the average fluorescence intensity. The manner of spot counting and area calculation showed about 20 min earlier in detecting the time. The results revealed the advantage in rapid detection by digitizing the counting method in array-LAMP.

## 4. Discussion

We demonstrated a plasmonic enhanced digitizing biosensor for nucleic acid detection. The sensor was composed of a nanodisc array and Bst polymerase conjugated on the surface of the nanodiscs. The detection principle was based on plasmonic enhanced fluorescence. The target nucleic acid was amplified by modified loop-mediated isothermal amplification with fluorescence resonance energy transfer (FRET-LAMP) primers. It showed capability in singleplex or multiplex nucleic acid detection. Although the gentle slope of the detection time calibration curve found in our research suggested that FRET-LAMP is more suitable for non-quantification screening, FRET-LAMP would still be very useful for fast and combination examinations for different viruses.

While integrating the plasmonic enhancement technology, the single-spot LAMP results indicated the contrast of FRET-LAMP was up to 7.4-fold to reject the background fluorescence from randomly unwound primer pairs and increase the signal-to-noise ratio. Further integration with a nanodisc array improved the detection time more. A nanodisc with a rough surface has two to four times the enhancement in the electrical field compared to a structure with a perfect flat surface. The hot spot generated on the surface contributed to the sensitivity of this nanoarray biosensor.

According to the results, less than 40 min was needed for detecting 10^−3^-μg/μL nucleic acid by FRET-LAMP, while the conventional LAMP control experiment with the same reaction conditions required 50–60 min. This result is comparable to the current work based on plasmonic enhanced Raman detection for the hybridization assay [5] and gold nanorod array chip [43]. Recently, a lot of digitizing LAMP assays based on microfluidics or droplets have been launched [17,44,45]. Most of them focus on the precise quantification of the analyte number. Due to lacking analyte isolation, the advantage of our design is in rapid detection instead of precise quantification.

Optimization of the reagent composition and reaction temperature has not been carried out yet in our experiment. Hence, a better performance would be expected with further optimization. In addition, variant active spot intensities were found in our FRET array-LAMP, which might imply that an uneven number of reactions happened at each spot. The optimization in controlling the polymerase number of each spot or in producing consistent nanostructures is important. A more precise fabrication technology like E-beam lithography, photolithography or nanoimprinting could be applied to ensure the sensor reproducibility in large-scale production (Figure S4).

In summary, the method we demonstrated in this work has shown the potential of a LAMP nanodisc array in developing high-contrast, rapid and multiplex nucleic sensing. We believe that an amplification integrated signal enhancement biosensor could be a useful technology in infectious pathogen screening in the future.

## 5. Conclusions

We demonstrated a new strategy for time lapse LAMP monitoring through the integration of plasmonic enhanced fluorescence, a nanodisc array, FRET-LAMP and a digitizing analysis. The main contribution of this work was to demonstrate a digitizing LAMP assay in a simple and high-signal contrasting manner without microfluidic isolation. Although the design did not show the same capability in counting the template number as other digitizing sensors, it proved to have rapid detection within 10 min, which is 20–30 min earlier than conventional LAMP under the same analyte concentrations. We expect this technology could be further applied to different pathogen DNA screenings in the future for rapid disease detection.

## Figures and Tables

**Figure 1:**
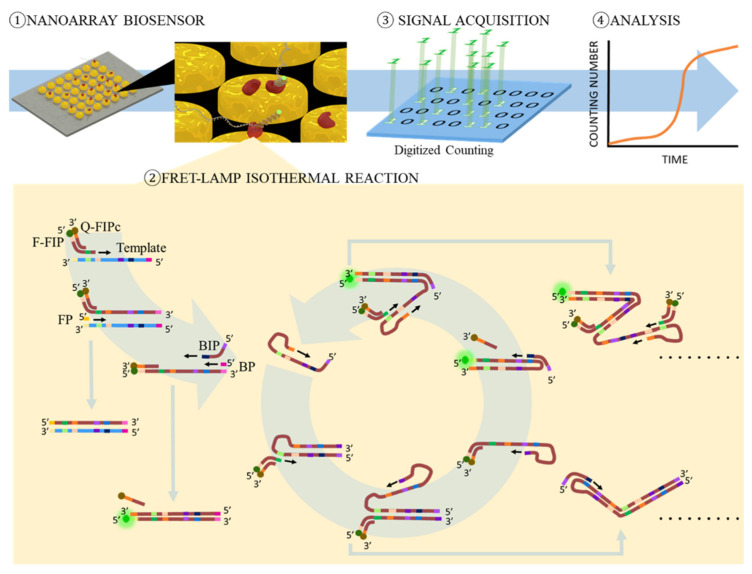
Step ①: Nanoarray was functionalized with Bst polymerase for FRET-LAMP. Step ②: A FRET FIP primer pair was used in FRET-LAMP. The fluorophore of the forward inner primer (F-FIP) would emit fluorescence, while the complement quencher probe (Q-FIPc) was removed during replication. Step ③: The reaction would be observed with a fluorescence microscope for time lapse detection. The signal was analyzed in a digitizing manner. Step ④: The trend of the counting number was then recorded.

**Table 1:** The FRET FIP primer pair sequence for HBV and HCV FRET-LAMP.

Name	Modification	Sequence
HBV F-FIP	5′-TAMRA	5′-TGG AAT TAG AGG ACA AAC GGG TGC TGC TAT GCC TCA TCT-3′
HBV Q-FIPc	3′-NFQ	5′-CCC GTT TGT CCT CTA ATT CCA -3′
HCV F-FIP	5′-FAM	5′-TAT GGC TCT CCC GGG AGG GGT TGC CAT GGC GTT AGT ATG AGT-3′
HCV Q-FIPc	3′-NFQ	5′-CCT CCC GGG AGA GCC ATA-3′

## Data Availability

Not applicable.

## Supplementary Materials

The following supporting information can be downloaded at https://www.mdpi.com/article/10.3390/bios12020075/s1: Figure S1: The relationship between Au sputtering film thickness and tip apex width. Figure S2: The illustration of surface modification and biomolecule conjugation for tip-LAMP and array-LAMP. Figure S3: The verification of FRET LAMP reaction with gel electrophoresis. Figure S4: The SEM image of Dip-pen nanolithography fabricated nanoarray and the fluorescence image of FRET array-LMAP. A drifting of the pattern alignment might happen in DPN nanofabrication.
