# Supplementary material for: Rapid Detection of Virus Nucleic Acid via Isothermal Amplification on Plasmonic Enhanced Digitizing Biosensor
Source: Biosensors (Basel). 2022 Jan 28;12(2):75. doi: 10.3390/bios12020075 (PMC8869753; doi:10.3390/bios12020075)
Supplement: Supplementary file 1 [file biosensors-12-00075-s001.zip › biosensors-1550467-supplementary.pdf]

biosensors MDPI

Supplementary

Rapid Detection of Virus Nucleic Acid via Isothermal Amplification on Plasmonic Enhanced Digitizing Biosensor

Shih-Chung Wei [1,2], Chia-Chen Chang [3,4], Tsung-Liang Chuang [2], Kung-Bin Sung [1] and Chii-Wann Lin [1,2,5,*]

1 Institute of Biomedical Electronics and Bioinformatics, National Taiwan University, Taipei 10617, Taiwan; wshihchung@gmail.com (S.-C.W.); kbsung@ntu.edu.tw (K.-B.S.)
2 Institute of Biomedical Engineering, National Taiwan University, Taipei 10617, Taiwan; light02062005@gmail.com
3 Department of Medical Biotechnology and Laboratory Science, Chang Gung University,33302, Taiwan; chang@mail.cgu.edu.tw
4 Kidney Research Center, Department of Nephrology, Chang Gung Memorial Hospital, Taoyuan 33302, Taiwan
5 Biomedical Technology and Device Research Laboratories, Industrial Technology Research Institute, Hsinchu 31057, Taiwan
* Correspondence: cwlinx@ntu.edu.tw

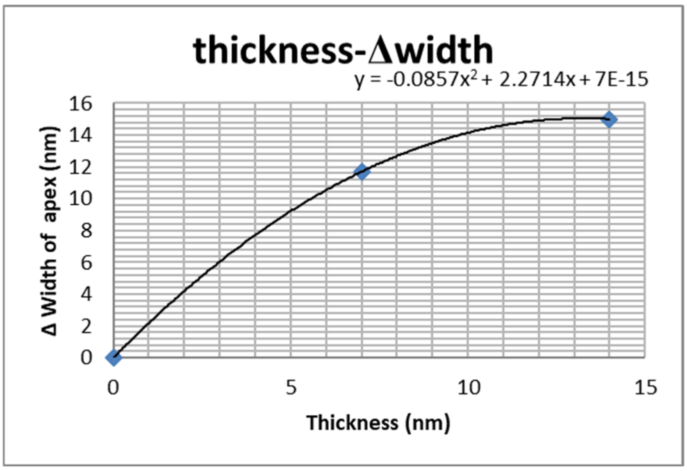

Figure S1. The relationship between Au sputtering film thickness and tip apex width.

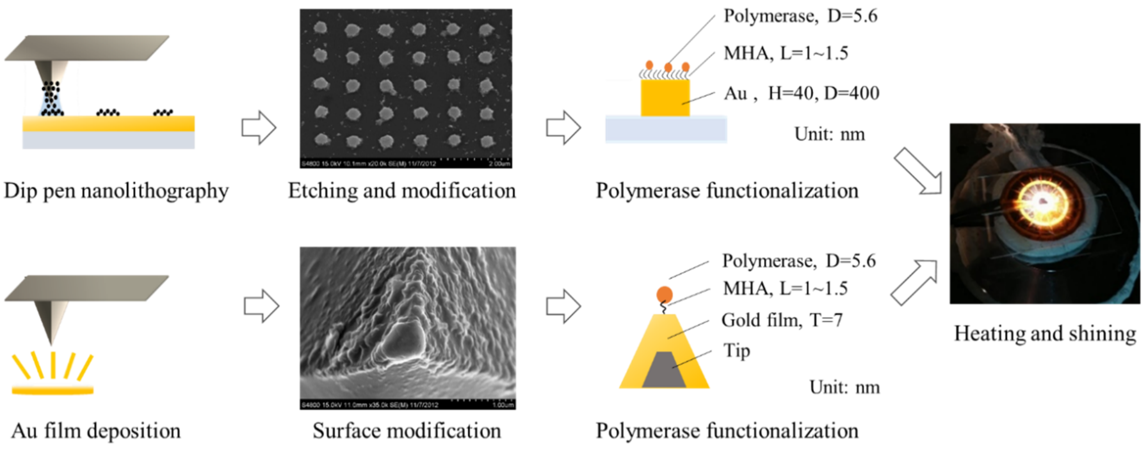

Figure S2. The illustration of surface modification and biomolecule conjugation for tip-LAMP and array-LAMP.

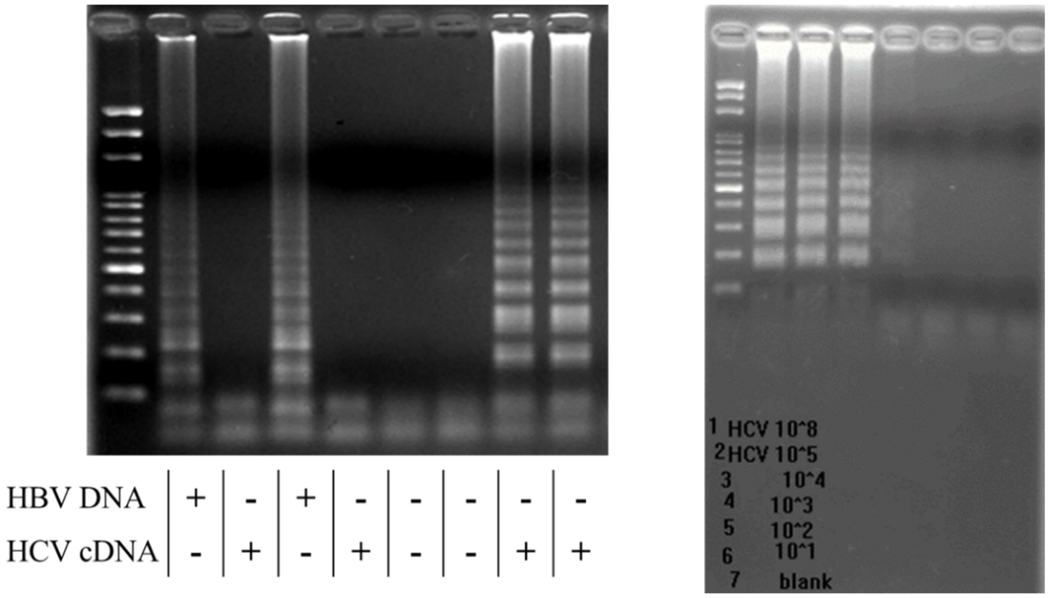

Figure S3. The verification of FRET LAMP reaction with gel electrophoresis.

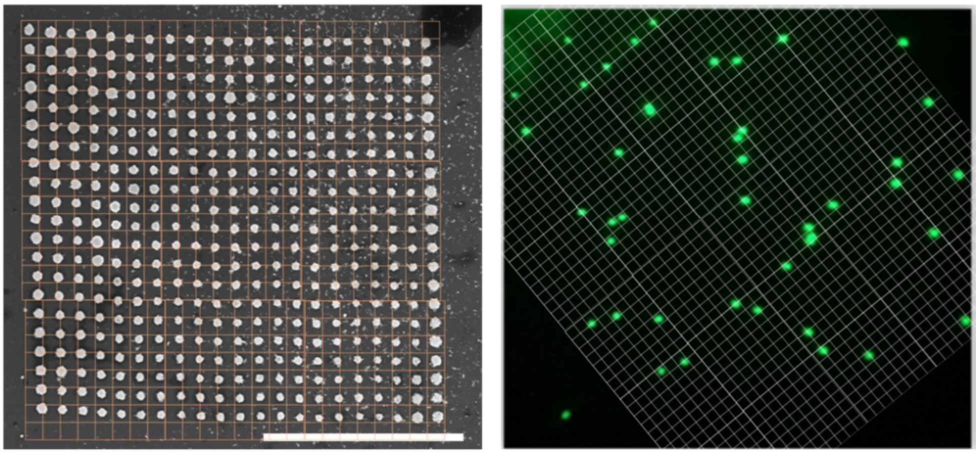

Figure S4. The SEM image of Dip-pen nanolithography fabricated nanoarray and the fluorescence image of FRET array-LMAP. A drifting of the pattern alignment might happen in DPN nanofabrication.
